# Interstitial Lung Diseases and Lung Cancer: A Review on Similarities, Common Pathogenesis and Therapeutic Approach

**DOI:** 10.3390/jpm15050213

**Published:** 2025-05-21

**Authors:** Gioele Castelli, Elisabetta Cocconcelli, Giuliana Grimaudo, Irene Di Leo, Serena Bellani, Giordano Fiorentù, Giacomo Giulianelli, Nicol Bernardinello, Elisabetta Balestro, Paolo Spagnolo

**Affiliations:** 1 Respiratory Disease Unit, Department of Cardiac, Thoracic, Vascular Sciences and Public Health, University of Padova and Padova City Hospital, 35128 Padua, Italy; gioele.castelli@phd.veneto.it (G.C.); elisabetta.cocconcelli@aopd.veneto.it (E.C.); giuliana.grimaudo@studenti.unipd.it (G.G.); serena.bellani@studenti.unipd.it (S.B.); giordano.fiorentu@studenti.unipd.it (G.F.); giacomo.giulianelli@studenti.unipd.it (G.G.); nicol.bernardinello@unipd.it (N.B.); elisabetta.balestro@aopd.veneto.it (E.B.); 2 Division of Respiratory Medicine, Department PROMISE, “Paolo Giaccone” University Hospital, University of Palermo, 90133 Palermo, Italy; irene.dileo@community.unipa.it

**Keywords:** idiopathic pulmonary fibrosis, interstitial lung diseases, lung cancer, lung cancer treatment, chemotherapy, immunotherapy, palliative care

## Abstract

Interstitial lung disease (ILD) prevalence and survival are increasing due to improvement in scientific research together with clinical complications typical of advanced disease. Lung cancer (LC) is described as a possible event occurring in lung parenchyma in the context of fibrotic abnormalities that worsen patients’ prognosis. This growth of malignant cells on a fibrotic background has also been called scar-cinoma. For this reason, not only an early diagnosis but also personalized decisions on the best treatment approach should be considered for each patient in a multidisciplinary discussion, since in some cases chemotherapy or surgery could be detrimental for patients with pulmonary fibrosis. LC and lung fibrosis may share common pathogenetic mechanisms like an altered healing process in response to repeated tissue damage from environmental exposure in genetically susceptible individuals. Smoking history and air pollution together with mutations in telomere and surfactant protein genes lead to the production of cytokines and nitro derivatives in the microenvironment that facilitate the carcinomatous transformation during fibrogenesis. The evolution of LC therapy and the implementation of immunotherapy acting on targetable immune checkpoints have raised interest in evaluating ILD-LC actionable mutations. The main pathogenetic mechanisms, clinical presentations and treatment implications are presented in this review.

## 1. Introduction

Interstitial lung diseases (ILDs) include a group of different disorders characterized mainly by alterations of the pulmonary interstitium, specifically inflammation and fibrosis in the space between the epithelial and endothelial basement [1]. Although the pulmonary interstitium is the main site of the damage, the alterations can also be localized at the alveolar space, the bronchial system and vessels [2,3,4]. The increase in scientific knowledge and specialized respiratory physicians seems to have increased the number of patients diagnosed with lung fibrosis globally, requiring an updated classification system [5]. However, strong epidemiological studies are needed to completely understand the reasons behind the apparent increase in diagnosis worldwide [6]. According to the balance between inflammation and fibrosis, the international consensus statement classified ILDs in four main categories [3]. ILDs may, therefore, be idiopathic, secondary to systemic diseases, secondary to known exposures (drugs, environmental, working exposures, etc.) or associated with cystic patterns/airspace filling. An accurate diagnosis of ILD is essential for treatment-related decisions and advising on prognosis. Although in previous years invasive examinations such as bronchoscopy or surgical lung biopsy were thought to be the only way to reach a correct diagnosis, in more recent years the multidisciplinary team, including pulmonologists, radiologists and pathologists, has become the gold standard for obtaining a correct ILD diagnosis. In the appropriate clinical and radiological context, many studies suggest that a surgical biopsy does not add useful information. Based on this concept, Flaherty et al. diagnosed 70 per cent of idiopathic pulmonary fibrosis (IPF) with no further information needed from invasive procedures [7]. Clinical features in ILDs include shortness of breath, exercise intolerance and persistent dry cough, with oxygen desaturation during exertion and resting hypoxia in the later phases. In case of advanced disease, signs of pulmonary hypertension with right heart failure and lung cancer may occur and worsen the prognosis [8]. The prolonged survival of patients with IPF due to innovative treatments like antifibrotics has recently increased the incidence of lung cancer among this specific population [9,10]. Obviously, a fibrotic interstitial lung fibrosis associated with a concomitant lung cancer (LC) impacts negatively on survival [11]. For this reason, not only an early diagnosis but also personalized decisions on the best treatment approach should be considered for each patient in a multidisciplinary discussion, since in some cases chemotherapy or surgery could be detrimental. In fact, lobectomy and chemotherapy could cause serious complications, and they could sometimes even be lethal [9].

## 2. Incidence and Risk Factors for Lung Cancer in Interstitial Lung Diseases

**Figure 1:**
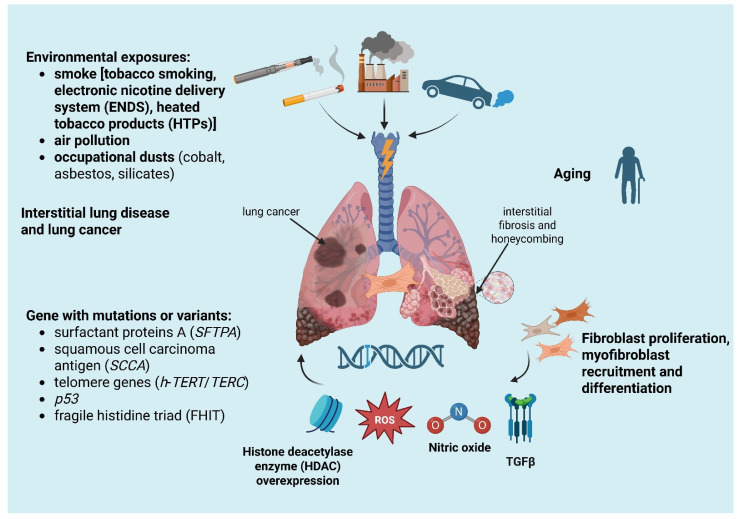
Main pathogenetic mechanisms implicated in the development of lung cancer among interstitial lung disease populations. Environmental exposures in the context of elderly patients affected by lung fibrosis promote lung injury and production of cytokines and nitro derivatives in the microenvironment that facilitate carcinogenesis in susceptible patients. Created in BioRender. https://BioRender.com/g53l545 (Accessed on 12 May 2025).

Patients with ILD have an increased risk of developing LC and consequently have a worse prognosis when compared to patients with lung cancer without interstitial lung disease. The association between lung fibrosis and lung cancer derives from common findings observed during autopsy of patients affected by lung cancer and fibrosis in the middle of the 20th century [12,13]. The relationship between ILDs and LC is a significant area of interest and concern in respiratory medicine. The association between the two conditions can be explained by common risk factors such as prolonged injuries, genetic predisposition, aging, environmental exposures, smoking history and common physiopathology of fibrogenesis and cancerogenesis. The relative risk (RR) for LC has been reported to be 3.5 to 7.5 times higher in ILD patients by Héluain and colleagues, with LC occurrence estimated at 10–20% in ILD [14]. When only IPF patients are considered, Gibiot et al. found an RR for developing lung cancer of 4.96 to 7.3 times higher than in control patients [15]. The association between IPF and lung cancer is even more stringent, perhaps due to the physiopathology of IPF that is directly related to epithelial damage, repair abnormalities and epithelial–mesenchymal transition. Otherwise, when the population affected by lung cancer is considered, ILD incidence is extremely variable, ranging from 2.4% to 24.3% [8,16,17]. In a recent meta-analysis, in IPF patients a high incidence of 2.07% per year was found, with LC causing death in 10% of IPF patients [18]. We can summarize the relationship between ILDs and LC considering the following mechanisms (Figure 1):

1. Occupational and environmental exposure: for example, cobalt exposure probably has a carcinogenic action, asbestos and silica exposure significantly increases the risk of small cell and non–small cell lung carcinoma, as well as causing respectively asbestosis and silicosis [19,20,21,22,23];
2. Smoking history: the relationship between cigarette smoking and ILDs is well known. In fact, cigarette smoking is the basis of the pathogenesis of desquamative interstitial pneumonia (DIP), respiratory bronchiolitis–interstitial lung disease (RB-ILD) and Langerhans cell histiocytosis. Smoking is also a common risk factor for LC and ILD, especially for IPF, causing a nine-fold increase in LC in former smokers and a twenty-fold increase in active smokers. However, even if typically strongly related to ILDs and LC, smoking is not always the main risk factor for the relationship between these two entities. For example, in systemic-sclerosis-associated ILD, LC does not have a higher prevalence in smokers than non-smokers and is probably more related to the inflammatory and immune processes of connective tissue disease [24,25];
3. Diffuse inflammatory process, present both in ILDs and in LC, includes epithelial abnormalities ranging from metaplasia to carcinomatous transformation, myofibroblast proliferation and soluble mediator release in the context of an abnormal healing process [16];
4. Gene alteration and aging with telomere attrition: mutations or variants in genes, for example, the gene for surfactant protein A (*SFTPA*) inducing tumor growth factor beta (*TGFβ*) secretion, squamous cell carcinoma antigen (*SCCA*), a serine protease inhibitor typically expressed by dysplastic/neoplastic cells of epithelial origin, abnormal telomere shortening (such as *h-TERT* or *h-TERC* mutation) and cellular senescence were found both in lung cancer and ILDs [26,27,28,29].

## 3. Pathogenesis of Lung Cancer in Interstitial Lung Diseases

Regarding the pathogenetic mechanisms shared by lung cancer and lung fibrosis, an altered healing process in response to repeated tissue damage and lung scar formation could probably predispose to lung cancer development [30]. Moreover, several reports suggest that, when fibrotic abnormalities are prevalent in interstitial lung disease, the risk for developing lung cancer increases [31]. Vancheri et al. stressed the concepts of aberrant proliferation, genetic alterations and tissue invasion of subepithelial lung fibroblasts in IPF in close association with cancer biology [32]. A direct relationship between fibrotic areas and cancer development, a phenomenon coined ‘scar-cinoma’, was suggested by the prevalent localization of lung cancer in the peripheral fibrotic areas of the lower lobes (56–90%) [9,33,34,35] and the predominance of tumor metaplasia on histological examination of fibrotic area in IPF patients [36,37,38], deriving from hyperproliferative preneoplastic lesions of the bronchiolar epithelium and lying within honeycomb cysts [39]. However, the process behind the development of LC in ILD is not yet fully known. In genetically predisposed individuals, repeated tissue injuries of different origin may lead to fibroblast activation, accumulation of extracellular matrix and abnormal bronchiolization of alveoli leading to honeycomb cysts. Furthermore, the interaction between epithelial cells with accumulated gene alterations and activated mesenchymal cells may trigger initiation and progression of LC. Importantly, epithelial and endothelial to mesenchymal transition significantly contribute to tumor metaplasia, invasion and metastasis [40].

The main processes involved in lung carcinogenesis secondary to an abnormal healing process are listed below:

1. Uncontrolled proliferation: the hyperplasia of pulmonary cells, both cuboidal and mucous cells, the evasion of apoptosis and an altered cell-to-cell communications appear linked to epithelial metaplasia and cancerogenesis. In lung tissue, transition zones from metaplasia to invasive cancer are located close to fibrotic areas [41];
2. Tissue invasion: myofibroblast recruitment and differentiation, with their ability to infiltrate tissues, together with invasive molecule expression are specifically linked to carcinogenesis. In pulmonary fibrosis, during myofibroblast differentiation Xie et al. observed the activation of the Warburg effect, a metabolic perturbation typical of cancer cells in which glycolysis is preferred over oxidative phosphorylation, even in the presence of oxygen [42,43];
3. Signal transduction pathways: the production of cytokines and nitro derivatives in the microenvironment facilitates carcinomatous transformation during fibrogenesis [32]. In particular, TGFβ, involved in fibrogenesis and myofibroblast transformation, normally exerts an antiproliferative action on epithelial cells [44]. Takenaka et al. proved that in IPF-LC patients Smad4 expression levels were significantly lower than in LC alone, promoting a diminished growth inhibitory response to TGFβ [45]. Nitric oxide (NO) production by stressed epithelial cells, like the ones in ILDs, causes guanine nitrification in cellular DNA. Terasaki et al. observed a NO overexpression and guanine nitration, especially in IPF and squamous cell carcinoma (SCC), compared to a control population regardless of smoking history [46];
4. Histone deacetylase enzyme (HDAC) overexpression: HDAC overexpression has been observed in myofibroblasts and abnormal bronchiolar epithelium of IPF [47]. Of interest, HDAC catalyzes deacetylation of many non-histone proteins, such as tumor suppressor p53, resulting in inhibition of its proapoptotic activity [48].

## 4. Distribution and Histopathology of Lung Cancer in Interstitial Lung Diseases

When lung cancer occurs in the context of pulmonary fibrosis, the neoplastic lesion is mainly localized in the peripheral areas of the lung (83.9%) developing within or near fibrotic areas (68.1%) especially in the inferior lobes (58.7%) [16]. According to Kewalramani et al., in 29% cases the neoplastic lesions appear in marginal-fibrotic areas and in 13% of cases in extra-fibrotic areas [31]. From a radiological point of view, on chest computed tomography (CT) scans, LC is mostly localized within fibrosis areas (44.4% of cases) followed by lesions in adjacent areas [49] and nodes in fibrotic areas are more probably LC even if smaller [50].

Most tumors in the context of ILD appear as solid, rounded or oval lesions, with a median doubling time of 77 days. As reported by Naccache et al. those lesions typically appear well delimited, with spiculated contours and an air bronchogram is often visible [16]. Among IPF patients, lung cancer presents a preferential localization in the lower lobes [31], while in non-IPF ILDs there is no difference in terms of localization of the LC lesions compared to the general population without pulmonary fibrosis. According to a Japanese clinicopathological study, patients with LC showing a usual interstitial pneumonia (UIP) pattern have a prevalent lower lobe localization of lesions, following the typical UIP pattern distribution [39].

The histological definition of lung cancer has been divided into two major subgroups: small cell lung cancer (SCLC) and non-small cell lung cancer (NSCLC). The latter is further divided into different histological entities: SCC, adenocarcinoma (ADC) and large cell carcinoma. Other rarer subtypes are present in the possible differential diagnosis of primitive lung neoplasia, such as carcinoids, lymphomas and sarcomatoid lesions [51].

Historically, in ILD-LC the most frequent tumor histotype is SCC. This is especially true in patients presenting a UIP pattern both in IPF and non-IPF ILDs [10,52,53,54,55]. This histotype has been associated with smoking history, partially explaining the higher prevalence in ILD patients. In IPF in particular, genetic predisposition seems to have a role only in this subtype of NSCLC [56]. The SCC diagnosis has fewer therapeutic options, especially in the context of ILDs, where immune checkpoint inhibitors may cause an exacerbation of the disease.

In more recent cohorts ADC has become the prevalent histotype of NSCLC in ILD populations [57,58,59]. This trend is especially true in patients presenting a non-UIP pattern, where the LC localization is predominantly in the upper lobes or in fibrosis-free parenchyma [60]. The shift towards ADC diagnosis is in line with the general population [61]. The reasons behind the histotype shift have been explained with the reduction of tobacco consumption and an increase in air pollution [62]. ADC necessitates further evaluation compared with SCC due to the possibility of target therapies in the case of specific genetic driver mutations, as presented below.

Regarding large cell lung cancer, data on its association with ILDs are scarce due to the rarity of the two.

SCLC, on the other hand, is present in a considerable percentage of ILD-LC patients, ranging between 10 and 25% of the cohorts [16]. The copresence of the two diseases is associated with higher mortality and chemotherapy side effects, especially in patients with more advanced fibrosis [63,64]. Synchronous cancers are more frequently described in IPF patients than non-IPF-ILDs [34]. Early stages are prevalent in non-IPF-ILD-LC, whereas advanced stages prevail in IPF patients or generally in the context of a UIP pattern [49].

## 5. Genetic Mutations: Similarities and Common Pathways

In recent years, genetic modifications and molecular expression of carcinogenic proteins have raised the interest of the scientific community. The research on lung tumors mainly focused on finding driver mutations actionable for therapies. In the context of ILD-LC, correlation and differences in the microenvironment and genetic landscape in patients with and without ILD have also been studied. Due to epidemiological prevalence, the majority of data presented are on LC in IPF.

The strongest relation found between lung cancer and lung fibrosis regards the mutations in surfactant protein A (*SFTPA*) genes. These mutations impair protein secretion leading to a familiar form of ILD, typically around the age of 45 years [65]. Also, rare variants, in particular in *SFTPA2*, are associated with the development of ILD [66,67]. In families presenting *SFTPA* mutations, the incidence of LC, with or without association with ILD, is augmented. Wang et al., using genetic linkage, found two rare missense mutations in the *SFTPA2* gene among two families affected by ILD and lung cancer at a young age. In this population, 10 patients presented ILD, 4 with concomitant LC and 3 other relatives presented isolated LC [26]. Another French study, starting from ILD patients with a personal or familiar history of LC, found a large family carrying a heterozygous *STPFA1* missense mutation [68]. Furthermore, mutations in the *SFTPA1* and 2 genes are not the only association between those genes and an increased risk of ILD and LC. An evaluation of the *SFTPA1-2* genetic variants in ILD patients from the OrphaLung network found a strong correlation with the development of LC [69]. Interestingly, the correct expression of *SFTPA1* may be a positive prognostic biomarker in lung ADC treated with immunotherapy due to the augmented presence of immune infiltrate associated with the presence of surfactant protein A1 [70].

Mutations in telomere-related genes (TRGs), such as *TERT, RTEL1, PARN* and *TERC*, are the main known risk factor for ILD development [71,72]. Those mutations, typically transmitted with an autosomal dominance, are associated with 25–35% of the familial forms of ILDs. TRG mutations are related to shorter telomeres and lead to ILD development around 60 years of age. Telomere shortening may also cause liver fibrosis and hematological diseases such as thrombocytopenia [73]. The correlation with LC, however, seems to be more complex. A large meta-analysis by Ma et al. found a correlation between shorter telomeres and LC [74], while a Mendelian randomization study found that longer telomeres seem to be associated with several neoplastic diseases, with lung ADC among them [75]. The expression of *hTERT* in LC samples seems not to differ when concomitant IPF occurs. However, *hTERT* seems to be expressed differently in IPF-LC than in LC without ILD, with a stronger nuclear expression in the IPF samples [76]. Interestingly, genetic variants of TRG seemed to be less related to the development of LC in the context of ILD than surfactant-related variants [69].

As for other genetic mutations studied, *TP53* seems to have a role both in fibrosing diseases and LC. This oncosuppressor gene is typically mutated with loss of function in several neoplastic lesions. Takahashi et al. reported multiple point mutations of *TP53* both in IPF-LC and IPF alone when the protein was overexpressed, relating these mutations to the inflammatory environment of fibrosing diseases [77]. This finding corroborated a previous study which found a high prevalence of *TP53* alterations in the peripheral zone of the fibrosis [78]. The high *TP53* mutation prevalence in the ILD-LC population has also been confirmed in a French population comprehending IPF and other ILDs [79]. TP53 alterations have also been found both in IPF fibrotic tissue and associated LC tissues. However, none of the alterations found were overlapping between LC and fibrotic tissue [80]. Another studied gene is the fragile histidine triad (*FHIT*), one of the most frequently altered genes in human neoplasia. *FHIT* has a role as a tumor suppressor gene promoting apoptosis and preventing epithelial–mesenchymal transition [81]. Uematsu et al. reported that allelic loss of the *FHIT* gene is involved in carcinogenesis in IPF patients, observing an increased rate of this mutation in IPF-LC patients over IPF without LC [82]. Modifications have also been found in the JAK-STAT pathway. STAT proteins, when phosphorylated, translocate to the nucleus to modify the expression of genes for cell proliferation or differentiation. Focal amplifications of STAT2 and STAT6 were identified in patients with IPF-LC with a possible role in the oncogenic pathway [62,83]. As confirmation of this carcinogenic genetic landscape, Demopoulos et al. found a large mutational burden in several tumor-suppressing genes, comprehending *FHIT* and *TP53*, in IPF blood and sputum samples [84].

## 6. Molecular Characterization of Lung Cancer in Interstitial Lung Diseases

As cited above, the evolution of lung cancer therapy and the implementation of tyrosine kinase inhibitors (TKIs) and immunotherapy raised interest in evaluating ILD-LC actionable mutations and PD-L1 expression. ILD-LC patients seem to have a low number of actionable mutations, consistent with the smoking history of those patients. For example, epithelial growth factor receptor (EGFR) mutations, the most common mutations in lung ADC, are rarely detected in LC-ILD. Five Japanese series evaluating *EGFR* gene mutations in non-small cell lung cancer (NSCLC) detected them in 0–5.8% of ILD patients vs. 24.3–47% of those without ILD [17,85,86,87,88]. On the other hand, some rare mutations seem to be more expressed in ILD-LC patients. Hwang et al. studied 35 surgically resected LCs in patients with early-stage ILD or good functional status matched with non-ILD resected LC. The presentation of LC in this population differed from the usual lower lobe presentation of ILD-LC. However, the majority appeared to be peripheral and near or inside the fibrotic and honeycombed area. The ADC subtype exhibited significantly fewer targetable mutations. They found that the *BRAF* gene was significantly more mutated in IPF-LC (17.1% vs. 2–4% in the general LC population reported in the literature). The study found an equal distribution of *BRAF* mutations between ADC and SCC subtypes. Moreover, all patients had non-p.V600E mutations, the most common *BRAF* mutation known [83]. Guyard et al. described 31 tumor samples collected from 18 IPF patients and 13 patients suffering from other lung fibrotic disorders (CTD-ILD, NSIP, pneumoconiosis and drug-induced lung fibrosis). The authors studied over 500 hotspot mutations in 22 colon and lung-cancer-associated genes with next generation sequencing and immunohistochemically evaluated ALK, ROS and PD-L1. In this population a high rate of *TP53* mutation was confirmed. No mutations were found in *EGFR, ALK* or *ROS1*, while the population presented a higher rate of *MET* mutation (20% of SCC and 8% of ADC against the 1% and the 2–7% reported in the literature) [79]. Kojima Y et al. in a large evaluation of resected ILD-LC compared to LC confirmed a lower *EGFR* mutation rate in ILD-LC, with a low presentation of *ALK* and KRAS in both the populations [68].

PD-L1 is an immune checkpoint protein, interacting with its ligand PD-1 expressed by T-cells, which allow tumoral cells to avoid the antitumoral immune response. The PD-L1/PD-1 axis has been implicated in ILD pathophysiology. An overexpression of PD-1 in CD4+T lymphocytes and PD-L1 expression in fibroblasts appear to promote fibrosis [89]. Furthermore, PD-L1 expression on lung cryo-biopsy was higher in IPF patients when compared with other ILDs [90]. This was also confirmed in the mediastinal lymph node tissue, as IPF patients presented a higher expression of PD-L1 in this tissue compared to LC patients [91]. In contrast, even if few data are presented in the literature, PD-L1 expression in ILD-LC patients seems to be low. In fact, in the study by Guyard and colleagues all ADCs but one had more than 50% stained tumor cells [79]. In a smaller population Heluain and colleagues found only 34.7% of patients presenting PD-L1 positivity [14], while Fujimoto and colleagues found 60% of patients positive, but with a median expression of 1% and only on surgically resected specimens [92]. Also, in an IPF-LC French cohort the prevalence of PD-L1-positive samples was low (15%) [69]. The molecular characteristics of LC in ILD are summarized in Table 1.

## 7. Management and Diagnostic Approach of Suspected Nodules in Interstitial Lung Disease Patients

Lung cancer is a frequent comorbidity in ILDs, with a higher incidence and reduced survival in IPF compared to other ILDs [10,94]. The high risk of lung cancer demonstrated in IPF patients mandates undertaking close surveillance with annual HRCT to detect malignancy in an early phase [95].

However, in some cases LC is much harder to identify on radiological imaging of ILD patients since the fibrotic areas seriously interfere with the detection of lesions indicating LC [96,97]. Due to difficulties in detecting nodules in the context of fibrosis, performing repeated CT scans helps to identify lesions worthy of further diagnostic investigation, including positron emission tomography–computed tomography (PET-CT) scans and histological examinations. Despite this, LC is often diagnosed already at an advanced stage in the majority of patients, with a serious impact on their survival. Tumor histotype identification with molecular evaluation analysis is therefore essential for approaching personalized therapy. An adequate diagnosis of both LC and ILD leads to a better approach to both diseases and an overall better therapy for the patient [98].

Despite epidemiological evidence suggesting that patients with IPF have a risk nearly five times as high as that of the general population of developing lung cancer, currently, no commonly accepted recommendations exist for monitoring those with IPF, and disease management of lung cancer remains similar to that adopted for the general population. The current Fleischner Society’s guidelines suggest that nodules 4 mm in diameter should be reassessed at 12 months, nodules between 4 and 6 mm in diameter should be reevaluated within the next 6 or 12 months and nodules sized between 6 mm and 8 mm in diameter should be reevaluated within the next 3 or 6 months, then at 9 or 12 months and finally at 24 months if they remain stable. When nodules greater than 8 mm in diameter and with a low or moderate risk for being malignant are detected, a positron emission tomography/computed tomography (PET-CT) scan should be performed [99]. A negative PET-CT uptake suggests chest CT surveillance or non-surgical lung biopsy is needed, whereas when PET-CT uptake is moderate/high and no metastases are reported, the current guidelines recommend surgical lung biopsy and resection, radiofrequency ablation or stereotactic body radiotherapy. Considering the high incidence of lung cancer in IPF patients, a new diagnostic approach of solitary nodules in these patients has been proposed to perform chest HRCT once a year in all patients with IPF [34]. The finding of nodules less than 8 mm in diameter should be followed up with chest HRCT every 3–6 months and PET-CT is recommended only in case of their progression. If clinical assessment does not favor surgical intervention, chest CT surveillance is recommended in cases of low to moderate probability for malignancy and non-surgical biopsy in case of a high probability of malignancy [100].

Considering the overall fragility of these patients, obtaining a histological diagnosis can be very difficult. In case of suspicion of a tumor lesion on PET-CT, minimally invasive diagnostic procedures have been proposed, including CT-guided transthoracic needle biopsy (TTNB) for peripheral lesions or ultrasound-guided endobronchial transbronchial needle biopsy if pathologic lymph nodes (≥8 mm in diameter) are also present. Patients deemed unsuitable for biopsy or with advanced tumor lesions will need to be discussed in an oncologic multidisciplinary discussion to find an individualized approach even if further diagnostic procedures and mild therapeutic regimens (e.g., antifibrotic agents and palliative care) are not planned [34].

## 8. Surgical Treatment

A large retrospective analysis demonstrated higher mortality and lower survival of patients with IPF who underwent surgical treatment of non-small cell lung cancer compared with patients without IPF [9]. Indeed, surgical treatment is greatly impaired by difficult management of postoperative complications, in particular, the acute exacerbation of interstitial lung disease [101,102]. However, because of the presence of limited data about the natural history, being affected by IPF as a comorbidity is still not considered a contraindication to surgical resection of lung cancer. In support of this concept, several studies evaluated radiotherapy as an alternative treatment approach to lung cancer, but reported similar or even higher rates of acute exacerbation than those that occurred after a surgical resection among fibrotic patients [103,104,105,106,107,108,109]. Therefore, radiotherapy or radiofrequency ablation cannot be recommended as alternative treatments to surgery for patients with lung cancer who have IPF [110]. The retrospective study conducted by Koizumi and colleagues on the risk for acute exacerbation of fibrosis among three different surgical approaches showed that the incidence of acute events was lower after a video-assisted thoracic surgery (VATS) than after a thoracotomy or a sublobar wedge resection, even if not statistically significant [109]. Moreover, these patients are found to have shorter 5-year survival than patients with IPF and a significant risk of postoperative acute exacerbation, surgery-related morbidity and mortality [8,9,105,106,107,111], as well as higher risk of developing second postresection primary lung cancer [112].

A composite scoring system for the identification of preoperative surgery-related acute exacerbation individual risk has been recently suggested [113] based on reliable predictors of unfavorable outcomes as higher levels of KL-6, serum C-reactive protein and serum lactate dehydrogenase (LDH), lower values of forced vital capacity (FVC) and diffusing lung capacity for carbon monoxide (DLco) on spirometry, as well as male gender, age >75 years, IPF versus other ILDs, preoperative steroid use, exertion dyspnea (Hugh-Jones classification), history of exacerbations and the presence of a definite UIP pattern on CT [108,114]. In addition, several studies have shown that combined pulmonary fibrosis and emphysema (CPFE) syndrome represents an important risk factor for the development of lung cancer [97,106]; this evidence provides an explanation for the higher frequency of lung cancer in patients with CPFE compared to IPF [9,115]. The presence of CPFE also represents a negative prognostic factor for survival [97,116] because of the higher postoperative complications and postoperative mortality in those patients who undergo surgery [49,117]. Hence, CPFE syndrome limits the management of lung cancer in a significant proportion of patients, considering patients’ poor clinical condition and the lack of standard care [49]. In conclusion, the indications for a surgical lung cancer resection in IPF patients should be decided based on the prognosis of IPF, the stage of lung cancer and other comorbidities.

## 9. Chemotherapy, Immunotherapy and Radiotherapy

In patients with interstitial lung disease, chemotherapy and radiotherapy could be complicated by pulmonary toxicity and there is no consensus on the ideal strategy to adopt for patients with unresectable lung cancer. UIP pattern [101] and lower values of FVC represent independent risk factors for chemotherapy-related complications [102] such as rapid deterioration, pulmonary infections, leukocytopenia, neutropenia, thrombocytopenia, peripheral neuropathy, respiratory insufficiency and cardiovascular complications [96]. However, fewer chemotherapy-related complications have been found in carboplatin-containing treatment regimens. A small retrospective study reported more beneficial effects of carboplatin combined with weekly paclitaxel than other regimens in patients with IPF and advanced NSCLC [118]. Indeed, the study demonstrated relatively good survival within 1 year of follow-up in 64% of the population and a median survival time of about 15.9 months. This suggests that carboplatin chemotherapy combined with paclitaxel regimens for advanced NSCLC with IPF are indeed effective. The efficacy and safety of treatment with platinum agents plus etoposide as first-line chemotherapy for SCLC in ILD were also demonstrated [52]. However, with regard to irradiation treatment, this seems to be more detrimental than beneficial for a significant proportion of patients with IPF [96]. Recent studies support the addition of antifibrotic drugs to chemotherapy regimen in patients with IPF and lung cancer. The therapeutic efficacy of the combination of pirfenidone with cisplatin compared to chemotherapy alone has been demonstrated [119] and the prophylactic effect of pirfenidone for postoperative exacerbations in patients with lung cancer and IPF has also been assessed [120,121,122]. Moreover, there is also evidence on the beneficial effect of nintedanib on the outcome of second-line docetaxel-based therapy, particularly for patients with ADC [95,123,124,125,126]. In the literature, some cases in which nintedanib has been used as a single agent to prevent the progression of IPF in the context of SCC or ADC have been reported [127,128]. Kai et al. even described a case of a patient with IPF who, after 7 months of NSCLC treatment with nintedanib alone, showed regression of the primary tumor, pleural dissemination and lymph node metastasis, as well as the absence of progression of IPF [129]. Nintedanib monotherapy has been proposed as an alternative treatment option for NSCLC in patients with IPF who cannot tolerate chemotherapy well [130]. The recent CASPIAN and IMpower133 studies proved the usefulness of adding an antibody blocking the PD-L1 molecule (atezolizumab in IMpower133 and durvalumab in CASPIAN) to the first-line SCLC chemotherapy based on platinum and etoposide [131]. Unfortunately, the efficacy on survival rates associated with the addition of atezolizumab or durvalumab to chemotherapy was not observed in NSCLC. Indeed, the high incidence of severe drug-related pneumonia in NSCLC patients with IPF comorbidity led to the interruption of the phase II TORG1936/AMBITIOUS study on the efficacy of atezolizumab in these patients [132]. A systematic review of studies presenting ILD-LC patients also found out that immune checkpoint inhibitors may have a better response in ILD patients, however, the same population suffered more and more intense immunotherapy-induced pneumonitis [133]. It has been described that immunotherapy (firstly with pembrolizumab and atezolizumab) has induced pneumonitis in a case of a patient with IPF and NSCLC and the use of nintedanib during the administration of atezolizumab therapy has been shown to ensure stabilization of drug-induced pneumonia without exacerbation [134,135]. Furthermore, other anti-VEGF drugs, such as bevacizumab, have been found to protect patients from chemotherapy-induced AE-ILD [136,137].

## 10. Palliative Care and End of Life Communication

The focus on clinical and surgical treatment should not overcome the psychological well-being of patients. Several studies underlined the importance of a palliative care approach in ILD patients and how this is an unmet need in most cases [138,139]. In the context of such heavily burdened diseases (ILDs and lung cancer), the correct communication with the patient and caregivers is of utmost importance [140]. Different strategies have been studied to plan palliative treatment and patients’ ‘end of life’ in oncological diseases and in particular LC. Advanced care planning has been proposed and means a punctual conversation about end-of-life treatment planning. However, a large multicentric trial showed that advanced care planning did not improve the quality of life in the patients included [141]. On the other hand, a more longitudinal approach, with several encounters over several months, demonstrated better planning and understanding of the patients, with an improved quality of life [142]. The best results on the symptomatic and psychological well-being of terminally ill patients have been reached with an early intervention of the palliative care team, alongside a better understanding of the disease by the patients [143,144,145,146].

The need to better communicate about the end of life and the activation of early palliative care is even greater in ILDs [147]. The prognosis of these rare diseases is poorly understood by patients, caregivers and probably even their general practitioners, and it is often difficult for clinicians to communicate the consequences of this disease condition [148]. Few studies have focused on the evaluation of the end-of-life period of these patients. In general, ILD end of life is burdened by a high healthcare cost, correlated with admission to high-dependency units and life-prolonging treatments [149,150]. Also, in ILDs alone, the early involvement of the palliative care team, accompanied by a multidisciplinary collaborative approach, showed a better result regarding quality of life in the advanced phase of the patient’s life [151,152,153]. Correct communication and preferably earlier end-of-life planning in the disease course are essential in ILDs due to the risk of life-threatening episodes like the acute exacerbations.

Palliative treatments in patients with ILD and LC do not differ and should be similar in patients presenting both diseases. They focus on the correct usage of opioids and oxygen therapy to control pain, dyspnea and cough [154,155,156]. Refractory dyspnea typical of end-stage ILDs could also benefit from non-pharmacological interventions such as fan therapy, exercise programs and pulmonary rehabilitation [154]. The severe hypoxemia associated with end-stage ILDs has demonstrated a better outcome when treated with high-flow nasal cannula oxygenation devices compared to conventional oxygen therapy or more invasive approaches [157].

## 11. Conclusions

The better characterization and earlier diagnosis of patients affected by pulmonary fibrosis and the prolonged survival offered by antifibrotics increase the incidence of other comorbidities such as lung cancer among patients with interstitial lung disease. Patients affected by lung fibrosis and concomitant lung cancer present a negative impact on survival. In the meantime, the evolution of lung cancer treatment approaches with the discovery of targetable immune checkpoints have changed the history of the treatment options offered to patients with lung cancer.

For this reason, both an early diagnosis and also a timely and personalized treatment are necessary for patients with lung cancer and concomitant lung fibrosis and to avoid severe and harmful complications.

## Figures and Tables

**Table 1:** Expression of targetable mutations and markers in ILD-LC samples.

Gene	Mutational Prevalence in ILD-LC	Mutation Prevalence in ILD-LC (%)	Mutation Prevalence in LC (%)	Citations
*EGFR*	Low prevalence	0.5–8%	24.3–47%	[17,85,86,87,88,89,93]
*ALK*	Low prevalence	0–1%	4%	[79,89]
*ROS1*	Low prevalence	0%	1–2%	[79]
*KRAS*	Low prevalence	8%	25%	[89]
*MET*	High prevalence	8–20%	2–7%	[79]
*BRAF*	High prevalence, especially non-V600E mutations	17%	2–4%	[83]
PD-L1	Rarely described, the expression of PD-L1 in ILD-LC seems to be low (median expression PD-L1 expression 1%, 37.5–85% not expressing PD-L1)			[14,69,79,92]

*EGFR*—epithelial growth factor receptor; *ALK*—anaplastic lymphoma kinase; *MET*—mesenchymal–epithelial transition (factor); *BRAF*—B-rapidly accelerated fibrosarcoma; PD-L1—programmed death ligand 1.

## Data Availability

Not applicable.
